# Is the routine health information system ready to support the planned national health insurance scheme in South Africa?

**DOI:** 10.1093/heapol/czab008

**Published:** 2021-04-02

**Authors:** Edward Nicol, Lyn A Hanmer, Ferdinand C Mukumbang, Wisdom Basera, Andiswa Zitho, Debbie Bradshaw

**Affiliations:** Burden of Disease Research Unit, South African Medical Research Council. South Africa; Division of Health Systems and Public Health, Faculty of Medicine and Health Sciences, Stellenbosch University, South Africa; Burden of Disease Research Unit, South African Medical Research Council. South Africa; Burden of Disease Research Unit, South African Medical Research Council. South Africa; School of Public Health, University of the Western Cape; Burden of Disease Research Unit, South African Medical Research Council. South Africa; School of Public Health and Family Medicine, University of Cape Town, South Africa; Burden of Disease Research Unit, South African Medical Research Council. South Africa; Burden of Disease Research Unit, South African Medical Research Council. South Africa; School of Public Health and Family Medicine, University of Cape Town, South Africa

**Keywords:** National Health Insurance (NHI), discharge summaries, routine health information system (RHIS), insurance claims, data quality, morbidity data, clinical coding, South Africa

## Abstract

Implementation of a National Health Insurance (NHI) in South Africa requires a reliable, standardized health information system that supports Diagnosis-Related Groupers for reimbursements and resource management. We assessed the quality of inpatient health records, the availability of standard discharge summaries and coded clinical data and the congruence between inpatient health records and discharge summaries in public-sector hospitals to support the NHI implementation in terms of reimbursement and resource management. We undertook a cross-sectional health-records review from 45 representative public hospitals consisting of seven tertiary, 10 regional and 28 district hospitals in 10 NHI pilot districts representing all nine provinces. Data were abstracted from a randomly selected sample of 5795 inpatient health records from the surgical, medical, obstetrics and gynaecology, paediatrics and psychiatry departments. Quality was assessed for 10 pre-defined data elements relevant to NHI reimbursements, by comparing information in source registers, patient folders and discharge summaries for patients admitted in March and July 2015. Cohen's/Fleiss’ kappa coefficients (*κ*) were used to measure agreements between the sources. While 3768 (65%) of the 5795 inpatient-level records contained a discharge summary, less than 835 (15%) of diagnoses were coded using ICD-10 codes. Despite most of the records having correct patient identifiers [*κ*: 0.92; 95% confidence interval (CI) 0.91–0.93], significant inconsistencies were observed between the registers, patient folders and discharge summaries for some data elements: attending physician’s signature (*κ*: 0.71; 95% CI 0.67–0.75); results of the investigation (*κ*: 0.71; 95% CI 0.69–0.74); patient’s age (*κ*: 0.72; 95% CI 0.70–0.74); and discharge diagnosis (*κ*: 0.92; 95% CI 0.90–0.94). The strength of agreement for all elements was statistically significant (*P*-value ≤ 0.001). The absence of coded inpatient diagnoses and identified data inaccuracies indicates that existing routine health information systems in public-sector hospitals in the NHI pilot districts are not yet able to sufficiently support reimbursements and resource management. Institutional capacity is needed to undertake diagnostic coding, improve data quality and ensure that a standard discharge summary is completed for every inpatient.

**KEY MESSAGES**

- This study provides a situational analysis of the level of preparedness of public-sector hospitals in South Africa for the planned implementation of the National Health Insurance (NHI) specifically in terms of claims reimbursements.
- The practice of completing discharge summaries as part of the patient-discharge process is suboptimal at all levels of public-sector hospitals in all provinces in South Africa.
- The absence of coded clinical data in many of these hospitals highlights the need for a national effort to support diagnostic and procedure coding, thus improving data quality to support the NHI reimbursements and resource management.
- Discharge summaries are often assessed for their role in ensuring patient safety and continuity of care but this study focuses on their implication for the planned NHI.

## Introduction

Universal Health Coverage (UHC) is one of the sustainable development goals and has been adopted as a key health policy goal by many countries including South Africa (Tangcharoensathien *et al.*, 2015). The aim is to improve access to quality essential health-care services and to safe, effective, quality and affordable essential medicines (United Nations, 2019). To achieve this, many countries such as the Philippines (Obermann *et al*., 2018), Ghana (Christmals and Aidam, 2020), Nigeria (Onoka *et al.*, 2015), Kenya (Abuya *et al.*, 2015) and Taiwan (Wu *et al.*, 2010) have implemented or are in the process of implementing a national health insurance (NHI) scheme, a financial system designed to pool funds at national level for the purchase of a package of services for all (Kutzin, 2012). Successful implementation of the NHI will require a well-functioning health information system.

South Africa has a well-established health-care system comprising public and private services operating in parallel. However, access to quality health care is uneven with acknowledged disparities based on wealth (Coovadia *et al.*, 2009; Harris *et al.*, 2011). As part of addressing these inequalities and ensuring UHC, South Africa is in the process of establishing an NHI scheme. A 2011 Green Paper (South African National Department of Health, 2011), followed by a White Paper (South African National Department of Health, 2015) established the intention to introduce the NHI. Ten NHI pilot districts were subsequently identified and given additional resources to start preparing for the NHI through a variety of focused interventions aimed at improving access and quality of care in the public sector (Matsoso and Fryatt, 2013). Piloting of the NHI is still in progress and plans are in place to scale up the scheme to other districts. As a part of these preparations, a Presidential Health Summit Compact, signed in 2019, identified the development of the information system to guide health-system policies, strategies and investments as one of the nine pillars to strengthen the South African health system (South African Government, 2019).

The demand for a morbidity-surveillance system to inform public-health actions as well as provide reliable health statistics is driven by the shift towards evidence-based approaches and demands for accountability (AbouZahr and Boerma, 2005). However, given South Africa’s plans to implement an NHI, the need for a standardized patient-information system has become urgent. Effective implementation of the NHI will require a patient-level data platform that can support reimbursement and resource management. At present, there is limited, if any, knowledge of the requirements for and the availability and quality of coded clinical data (mainly on diagnoses and procedures/interventions) to support hospital services in South Africa. Despite the recognition that reliable data will be required to support the implementation of the planned NHI, there has been no known systematic review of availability to date (Matsoso and Fryatt, 2013).

Concerning the plans to implement the NHI in South Africa (Matsoso and Fryatt, 2013), it is important to assess the suitability of current routine health information system (RHIS) practices in public-sector hospitals to support patient management as well as the funding of medical care. Although there is adequate data to inform the national burden of disease measurements focusing on mortality (cause of death) in South Africa (Pillay-van Wyk *et al.*, 2013, 2016), valid and comparable measurements of morbidity (cause of ill-health) to inform health-service management and planning remain problematic (Salomon, 2010). Current sources of patient-level morbidity information in South Africa are fragmented—in terms of coverage between and within health facilities and across the country—and incomplete (Mate *et al.*, 2009; Auld *et al.*, 2013; Rose *et al.*, 2013; Roomaney *et al.*, 2017). In common with other developing countries, platforms for primary data collection in health systems have not yet made full use of the developments in information technology, and data systems are generally less than ideal (Health Metrics Network, 2012). These challenges are related to the nature and quality of the RHIS (Ledikwe *et al.*, 2014; Nicol *et al.*, 2016; Nicol *et al.*, 2017).

The World Health Organization (WHO) designed the International Classification of Diseases (ICD) framework (WHO, 2019a), an alphanumeric code-based model to support the classification of medical diagnoses (Manchikanti *et al.*, 2011). These codes translate narrative documentation into concise terms that insurance companies use to understand medical diagnoses and treatment interventions to process and reimburse health insurance claims (Dyers *et al.*, 2016). ICD-10, the 10th revision of the WHO International Classification of Disease and Related Health Problems, is the current national standard for diagnosis coding in South Africa (South African National Department of Health, 2014; WHO, 2019a). Although ICD-11 has been published (WHO, 2019b), it is only due for implementation from 2022.

While the National e-Health Strategy (2012–16) appeals for standardized, coded clinical records as a component of health information systems to support effective health-services implementation (South African National Department of Health, 2012), the potential success of the NHI also necessitates a reliable, standardized patient-level health information system that supports Diagnosis-Related Groupers (DRGs) for reimbursements and resource management (Matsoso and Fryatt, 2013). To this end, discharge summaries, an overview of a patient’s hospitalization from admission through to discharge including information relating to the cause of morbidity and treatment following discharge, are critical.

Discharge summaries play a pivotal role in the information flow and provide vital information for the continuation of patient care following discharge and should contain information such as a patient’s identification, the reason for hospitalization, procedures performed, care, treatment and services provided, patient's condition and disposition at discharge, the information provided to the patient and family, and provisions for a future treatment plan (Regenstrief Institute inc., 1995; Kind and Smith, 2008). However, their usefulness depends on the quality of the underlying routine patient health records and how accurately such clinical information is transferred to the discharge summary (Health and Social Care Information Centre, Academy of Medical Royal Colleges, 2013). Different reviews have emphasized the importance of quality discharge summaries in the continuity of patient care (Karaksha *et al.*, 2010; Chin *et al.*, 2013), and have shown the quality of discharge summaries to be suboptimal (Van Walraven and Weinberg, 1995; Kind and Smith, 2008; Were *et al.*, 2009; Horwitz *et al.*, 2013; Al-Damluji *et al.*, 2015).

The South African National Health Act (South African National Department of Health, 2004) requires that written discharge summaries are provided to all in-patients at the time of discharge and that copies should be kept in the patient’s health record. While discharge summaries are important for the follow-up and clinical management of patients (Callen *et al.*, 2008), they are also used to inform insurance billing (Manchikanti *et al.*, 2011). The diagnosis and procedures received by each patient must therefore be accurately identified and classified.

Despite the importance of discharge summaries as a source of morbidity data and their potential usefulness for the successful implementation of the NHI, there are a dearth of studies in resource-limited settings examining the availability of discharge summaries and codable clinical data and the quality of patient health records. This study was designed to assess: (1) the quality of patient health records; (2) the congruence between the information recorded on the discharge summaries and patient health records; and, (3) the availability and completeness of standard discharge summaries in public-sector hospitals in South Africa.

## Methods and materials

### Study setting

**Figure 1:**
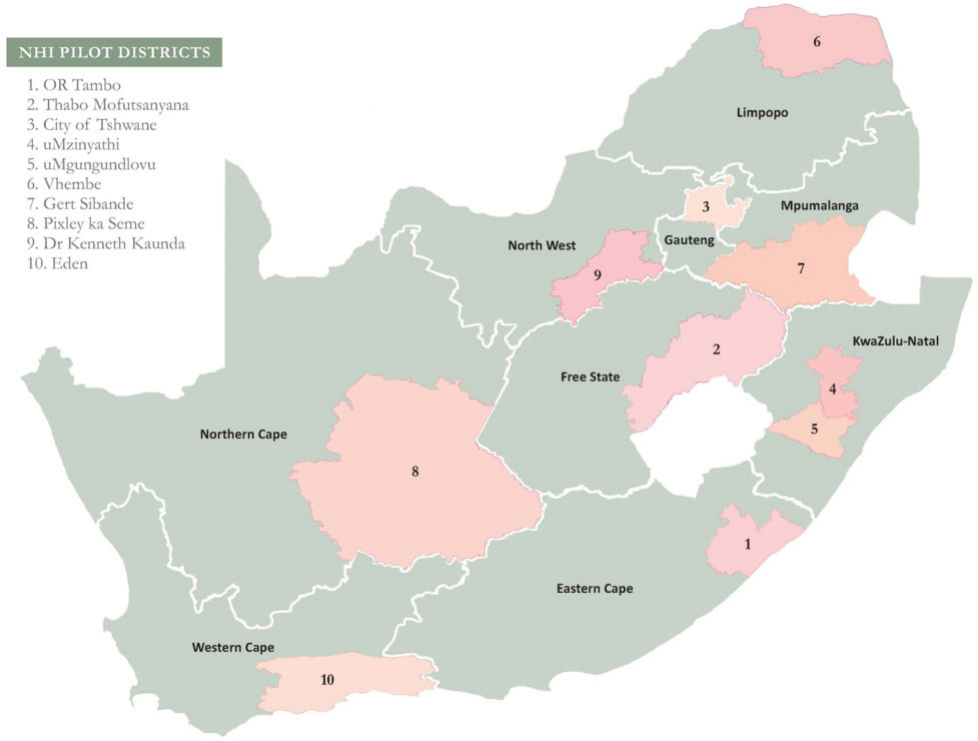
NHI pilot districts. 1. OR Tambo; 2. Thabo Mofutsanyana; 3. City of Tshwane; 4. uMzinyathi; 5. uMgungundlovu; 6. Vhembe; 7. Gert Sibande; 8. Pixley ka Seme; 9. Dr Kenneth Kaurnda; 10. Eden.

A sample of public-sector hospitals across 10 NHI pilot districts selected by the South African National Department of Health (NDoH), was identified (Figure 1). These districts were selected based on a combination of factors such as demographics, socio-economic factors including income levels and social determinants of health, health profiles, health-delivery performance, health-service management, financial and resource management (Matsoso and Fryatt, 2013).

### Study design

A retrospective cross-sectional health records review was undertaken on a sample of public-sector hospitals in South Africa, with a focus on addressing all health-systems bottlenecks and challenges to reverse the worsening disease burden. The NHI pilot sites were established to test the feasibility of implementing the NHI to reduce the high maternal and child mortality rates in South Africa and other components of the disease burden. The objectives of the pilots include testing the ability of the districts to assume greater responsibilities under the NHI, to assess utilization patterns, and costs and affordability of implementing a primary health-care service package (Matsoso and Fryatt, 2013).

### Study population

The sampling frame for the study was all public hospitals with their five treatment departments—surgical, medical, paediatrics, obstetrics and gynaecology and psychiatry, located within the 10 NHI pilot districts (*N* = 83).

### Sampling technique

A cluster study design was adopted whereby each of the NHI pilot districts was considered a cluster.

#### Sample size

To cover each stratum (i.e. hospital level), proportional sampling was used to randomly select three district hospitals, and all regional and tertiary hospitals from each of the pilot districts in the nine provinces to yield a total of 45 hospitals. Table 1 outlines the breakdown of the sampled hospitals. The sample size of in-patient health records was determined by assuming a 50% prevalence for the number of admissions per hospital, with a 95% confidence level and a precision level of 0.05. Given the cluster design of the study and unknown effect on the data, a design effect of 1.5 was assumed. Based on these parameters, a sample of 578 in-patient records was estimated for each district.

**Table 1:** Characteristics of the different hospital levels (South African National Department of Health, 2004)

Care level	Characteristics of hospital type	Total	Number sampled
Tertiary hospitals (Level 3)	Has between 400 and 800 beds Provides specialist services Provides intensive care services under the supervision of a specialist Receives referrals from regional and district hospitals without provincial boundaries	7	7
Regional hospitals (Level 2)	Has between 400 and 800 beds Provides specialist services on a 24-h basis Receives outreach support from tertiary hospitals	10	10
District hospitals (Level 1)	Serves a defined population within a district and supports primary health care. Can have from 50 to 600 beds depending on the size Provides district package of care on a 24-h basis General practitioners and clinical nurse practitioners providing health services. Provides in-patient, emergency and ambulatory health services.	51	28^a^
Total hospitals		68	45

a There were only two district hospitals in two of the districts (Dr K Kaunda and uMgungundlovu).

Consequently, data were expected from 5780 routine in-patient-level records at 45 sampled public-sector hospitals from five treatment departments or groups of departments—surgical, medical, paediatrics, obstetrics and gynaecology and psychiatry. For consistency across the hospitals, all departments in each hospital were assigned to one of these five groups. The records were drawn proportionally based on the estimated number of admissions in the selected hospitals during the study months to allow for seasonal disease surges (March 2015, summer season and a peak for diarrhoeal cases, and July 2015, winter period) and the number of hospitals per level in each NHI pilot district (Table 2).

**Table 2:** Estimated numbers of records for review by types of public hospitals within NHI pilot districts

	GP	WC	NC	NW	MP	FS	KZN1	KZN2	LP	EC	Total
Facility type	Tshwane	Eden	Pixley ka Seme	Dr K Kaunda	Gert Sibande	Thabo Mofutsanyana	uMgungundlovu	uMzinyathi	Vhembe	O R Tambo	
District (Level 1) hospital	3 (149)^a^	3 (388)	3 (578)	2 (85)	3 (506)	3 (390)	2 (222)	3 (578)	3 (430)	3 (260)	28 (3586)
Regional (Level 2) hospital	1 (64)	1 (190)	0 (0)	2 (336)	1 (72)	2 (188)	1 (255)	0 (0)	1 (148)	1 (95)	10 (1348)
Tertiary/central (Level 3) hospital	3 (365)	0 (0)	0 (0)	1 (156)	0 (0)	0 (0)	1 (101)	0 (0)	0 (0)	2 (224)	7 (846)
Total	7 (578)	4 (578)	3 (578)	5 (578)	4 (578)	5 (578)	4 (578)	3 (578)	4 (578)	6 (578)	45 (5780)

a Estimated number of folders in parenthesis (Based on proportional sampling). GP: Gauteng Province; WC: Western Cape; NC: Northern Cape; MP: Mpumalanga Province; FS: Free State Province; KZN: KwaZulu-Natal Province; LP: Limpopo Province; EC: Eastern Cape Province.

Depending on the size of the hospital, approximately 10 records were accessed from each of the treatment departments for each study month, at each study hospital. All records were accessed if the number of admissions during a study month in a department was <10.

### Data-collection tools

Data were collected between August 2016 and April 2019 by trained fieldworkers. Research teams were given log sheets to be signed by the managers (CEOs) of the hospitals visited. The log sheet included the names of hospitals visited, time spent at the facility and date of visit. Also, the teams were given a data-collection summary checklist (Supplementary Appendix SA) outlining the data-collection activities conducted in each hospital. These included extracting and photographing information from selected in-patient health records contained in registers, patient folders and discharge summaries and extracting information from available eRHIS (electronic RHIS). This information was captured using the Research Electronic Data Capture (REDCap), a web-based application for building and managing online surveys and databases (Harris *et al.*, 2009).

The project manager reviewed the completed instruments and the data-collection summary checklist and communicated any inconsistencies to the supervisors/fieldworkers to resolve any data-quality problems that occurred during fieldwork. Document and documentation standards and the availability of discharge summaries were investigated using a data-collection checklist (Supplementary Appendix SA). This checklist was used to identify the relevant documents and information on the availability of patient-discharge summaries. The presence of a patient-discharge summary was confirmed by taking a de-identified photo of the record and uploading it onto REDCap.

### Data analysis and management

Record quality was measured using two dimensions: (1) Completeness of the data in the ward register, patient medical record and discharge summaries; and (2) data accuracy i.e. the agreement between data in the patient medical record (paper-based and electronic), discharge summaries and ward register for 10 pre-defined data elements: patient age, patient identifier, attending physician’s signature, admission diagnosis, discharge date, discharge (final) diagnosis, condition on discharge, procedures, follow-up plan and results of the investigation (Wimsett *et al.*, 2014).

Data completeness was assessed by reviewing the proportion of discharge summaries that had all the required data fields completed by a clinical registrar, a general practitioner/medical officer or nursing staff. A percentage average of the availability of coded diagnoses during the two 1-month periods was reviewed. Record accuracy was investigated at two levels by measuring the agreement analysis for the 10 pre-defined data elements:

- comparing whether the admission diagnosis, patient identifier, admission date and attending physician’s signature recorded in the ward registers matched what was recorded in the patient folder/medical report; and
- comparing the patient folder/medical record with the patient discharge summary for the 10 data elements.

Statistical analyses were completed using the ***svyset*** command in STATA 16.0 (StataCorp LLC,) to incorporate the three-stage cluster study design of the sample. For the first stage, the primary sampling unit was the hospital/facility stratified by the hospital/facility type and there was no finite population correction since the number of records to be reviewed was not known beforehand. The second and the third stages only had the study month and the facility departments as the primary sampling units, respectively. Once set, proportions for the documents and the documentation standards were estimated and reported with their respective 95% confidence intervals (CIs).

Cohen's and Fleiss’ kappa coefficients (*κ*) were used to measure intra-rater reliability between the values of the pre-defined data elements found in the discharge summaries compared to the patient’s health records and ward registers, ignoring the survey design. Cohen’s kappa was used for the 10 data elements for the two data sources—patients’ folder vs. discharge summaries and, where ward registers were included in the comparison, Fleiss’ kappa was used. We reported the measure of agreement/kappa scores and the CI ranges. A *P*-value of <0.05 eliminated chance agreement and CIs were also reported.

### Ethics consideration

The study proposal received ethics clearance from the Human Research Ethics Committee of the South African Medical Research Council (Ref: EC 003-2/2016) and the University of Pretoria Health Ethics Committee (Ref No: 305/2017). Permission to access the patient’s health records from the various hospitals was obtained from the respective provincial and district health departments, and the study hospitals. Because the study did not require direct interactions with patients, patient consent was not required. However, strict confidentiality was adhered to with regard to the protection of information obtained from patient records; individual patient health records were de-identified and assigned a unique subject identifier at the point of data collection. The record of the links between project ID codes (unique subject identifier) and the patient identifiers (folder numbers) was securely kept in an encrypted database (REDCap).

## Results

A total of 5795 routine inpatient-level records were reviewed at all 44 sampled public-sector hospitals from five treatment departments (Table 3). Most of the data were abstracted from the medical departments (27%, 1533/5795), while the psychiatry departments accounted for the least records (7%, 401/5795), abstracted from 61% (27/44) of the sampled hospitals.

**Table 3:** Overall response by facility type and treatment departments

Facility type	Medicine	Surgery	Paediatrics	Obstetrics	Psychiatry	Total
District (Level 1) hospital *n* (%)	1033 (29)	696 (20)	791 (22)	873 (25)	141 (4)	3532 (100)
Regional (Level 2) hospital *n* (%)	280 (20)	262 (18)	351 (24)	395 (28)	149 (10)	1437 (100)
Tertiary/Central (Level 3) hospital *n* (%)	220 (27)	223 (27)	137 (17)	135 (16)	111 (13)	826 (100)
Total *n* (%)	**1533** **(27)**	**1181** **(20)**	**1279** **(22)**	**1401** **(24)**	**401** **(7)**	5795 (100)

Table 4 gives an overview of the total responses by pilot district and province. Response rates were calculated for each pilot district and facility type using the sample size in Table 2 as denominators. The overall record response rate of 100.3% (5795/5780) shows that the results are representative of the target population based on the estimated numbers of patient health records in the NHI pilot districts (Table 2). However, the response rate for the primary sampling unit (i.e. the hospital) is 98% (44/45), due to one of the sampled hospitals (Ventersdorp Hospital in Dr K Kaunda district, North West) being downgraded to a Community Health Centre during the data-collection phase and therefore its exclusion from the study (Table 4).

**Table 4:** Response rate (Rr) showing overall proportion against the target by facility type, pilot district and province

	GP	WC	NC	NW	MP	FS	KZN1	KZN2	LP	EC	Total
Facility type	Tshwane	Eden	Pixley ka Seme	Dr K Kaunda	Gert Sibande	Thabo Mofutsanyana	uMgungundlovu	uMzinyathi	Vhembe	O R Tambo	
District (Level 1) hospital	3/3 (149)	3/3 (388)	3/3 (578)	1/2 (85)	3/3 (506)	3/3 (390)	2/2 (222)	3/3 (578)	3/3 (430)	3/3 (260)	27/28 (3586)
(n1/N, n2)											
District Rr (%, n3)	**98** (146)	**105** (409)	**100** (578)	**92** (78)	**95** (483)	**99** (389)	**93** (207)	**100** (580)	**97** (418)	**98** (256)	**99** (3544)
Regional (Level 2) hospital (n1/N, n2)	1/1 (64)	1/1 (190)	0 (0)	2/2 (336)	1/1 (72)	2/2 (188)	1/1 (255)	0 (0)	1/1 (148)	1/1 (95)	10/10 (1348)
Regional Rr (%, n3)	**106** (68)	**103** (196)	**-**	**100** (336)	**60** (43)	**107** (201)	**100** (256)	**-**	**115** (170)	**164** (156)	**106** (1426)
Tertiary/central (Level 3) hospital (n1/N, n2)	3/3 (365)	0 (0)	0 (0)	1/1 (156)	0 (0)	0 (0)	1/1 (101)	0 (0)	0 (0)	2/2 (224)	7/7 (846)
Tertiary Rr (%, n3)	**104** (378)	**-**	**-**	**114** (178)	**-**	**-**	**102** (103)	**-**	**-**	**74** (166)	**98** (825)
Total	7/7 (578)	4/4 (578)	3/3 (578)	4/5 (578)	4/4 (578)	5/5 (578)	4/4 (578)	3/3 (578)	4/4 (578)	6/6 (578)	44/4 5 (5780)
Total Rr (%, n3)	102 (592)	105 (605)	100 (578)	102 (592)	91 (526)	102 (590)	98 (566)	100 (580)	102 (588)	100 (578)	100 (5795)

n1, number of targeted facilities; N, total number of available facilities; n2, estimated number of folders; n3, number of folders obtained.

^a^ Dr K Kaunda district only has two district level hospitals, of which one was downgraded to a Community Health Centre and therefore no longer met the sampling criteria.

### Data on clinical details and quality assurance: documentation standards

Our first point of call was to assess the completeness of the patients’ folders in their entirety. The assessment was based on the study by Adeleke *et al.*, (2012), the audit tool by the Royal College of Physicians (2009) and a modified version of the WHO guide for improving data quality (WHO 2003), which included the content of a standard medical record (availability of clinical details) and documentation standards (related to types of documents that form part of the patient’s folder). The assessment of the clinical details in the folders showed that follow-up details are written as part of the discharge notes in 65% (3750/5789; 95% CI 55.5–74.0) of the patients’ folders and that 66% of the standard patient health records included discharge summaries (3884/5745; 95% CI 56.2–74.9) (Table 5).

**Table 5:** Document and documentation standards

Detail	***n*/*N*** ^b^	Percentage: 95% CI
Clinical detail and quality assurance—document standards		
Discharge notes are recorded in progress notes on discharge	4454/5790	77 (70.8–83.1)^a^
Follow-up details are written as part of the discharge notes	3749/5787	65 (55.4–74.1)^a^
Standard medical record includes a discharge summary	3767/5743	66 (56.1–75.0)^a^
Clerking and follow-up notes from admission to discharge	5145/5789	89 (84.7–93.1)
Discharge summary completed by a clinician	3695/3884	95 (91.1–99.2)
Data reliability, consistency and responsibility for care—documentation standards		
All pages contain patient’s full names	3722/5788	64 (55.6–73.0)^a^
Patient identifier recorded in all pages	3607/5790	62 (53.8–70.8)^a^
All pages contain correct patient identification	3664/5791	63 (54.5–72.0)^a^
Progress notes from admission to discharge	4926/5789	85 (80.4–89.8)
Progress notes documented daily	4939/5789	85 (80.6–90.0)
Notes signed and dated daily	5024/5789	87 (82.5–91.0)

a Elements that did not meet the exception rate of ±20% tolerance levels (i.e. the permissible range of variation) within expected values.

b Variation in *N* (denominator) as a result of missing data.

Concerning documentation standards, only 64% (3722/5788; 95% CI 55.6–73.0) of the folders had patients’ names on all pages, 62% (3607/5790; 95% CI 53.8–70.8) had patient identifiers and 63% (3664/5791; 95% CI 54.5–72.0) contained correct patient identification. Important to note is also the fact that 95% (3695/3884; 95% CI 91.1–99.2) of the discharge summaries were completed by the attending clinician. Considering that writing the discharge summaries is the responsibility of the attending clinician, it is problematic that some of the discharge summaries were not completed by them. All the other elements met the requisite standards as illustrated in Table 5.

### Availability of discharge summaries

**Figure 2:**
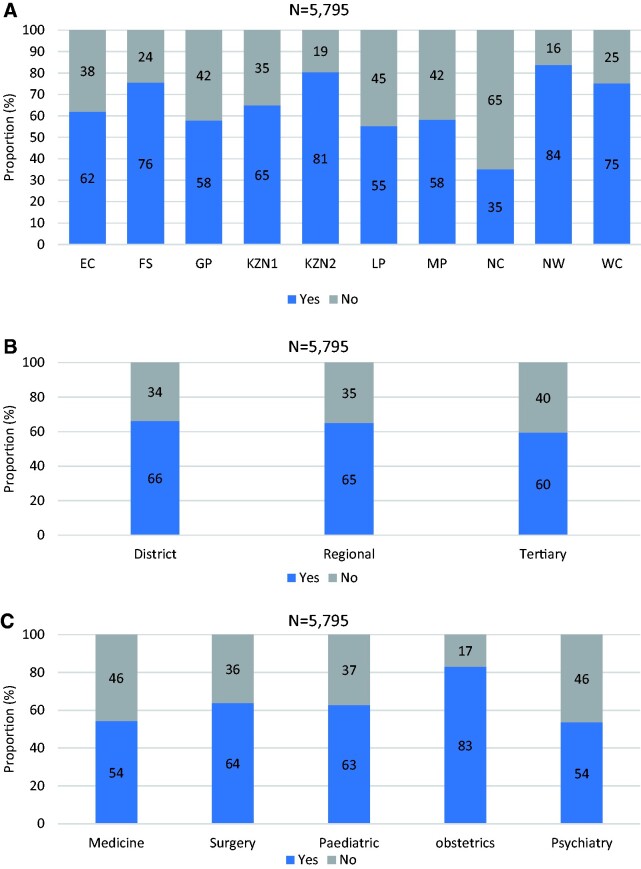
(a) Proportion of available discharge summaries by NHI pilot district. (b) Proportion of available discharge summaries by hospital type. (c) Proportion of available discharge summaries by hospital department.

Our analysis revealed that only 65% (3768/5795) of the inpatient health records in the study hospitals contained discharge summaries with 0.9% (52/5795) having missing information. At the provincial level, the pilot districts in the North West province performed best with 84% (496/592) of patients receiving discharge summaries, while the pilot districts in the Northern Cape province at 35% (203/578), were the worst-performing pilot districts (Figure 2a). Regarding the type of hospital, tertiary hospitals (60%; 491/825) performed slightly worse than district hospitals (66%; 2346/3542) and regional hospitals (65%; 930/1428) (Figure 2b). At the department level, obstetric departments showed the best practices regarding discharge summaries (83%; 1164/1401) with the medical (54%; 832/1533) and psychiatric (54%; 215/401) departments showing the worst practices (Figure 2c). The observed differences in performance in terms of availability of discharge summaries at the province level, type of hospital and hospital department were all statistically significant (*P* < 0.001).

### Availability of coded or codable data

The findings show that despite the availability of codable clinical data in inpatient health records, only 15% (835/5575) of the records contained any diagnosis data coded using the ICD-10 classification, which is the South African national standard for diagnosis coding. For those records with a discharge summary, 14% (529/3759) included diagnoses coded using the ICD-10 classification. Three pilot districts in three provinces (Eastern Cape, Free State and KwaZulu-Natal, i.e. uMzinyathi District) did not have any diagnoses coded in their patient’s health records regardless of existence or absence of discharge summaries, and the pilot district in the North West province only had 0.5% (3/592) and 0.6% (3/496) of diagnoses coded in the patient folders and discharge summaries, respectively.

**Figure 3:**
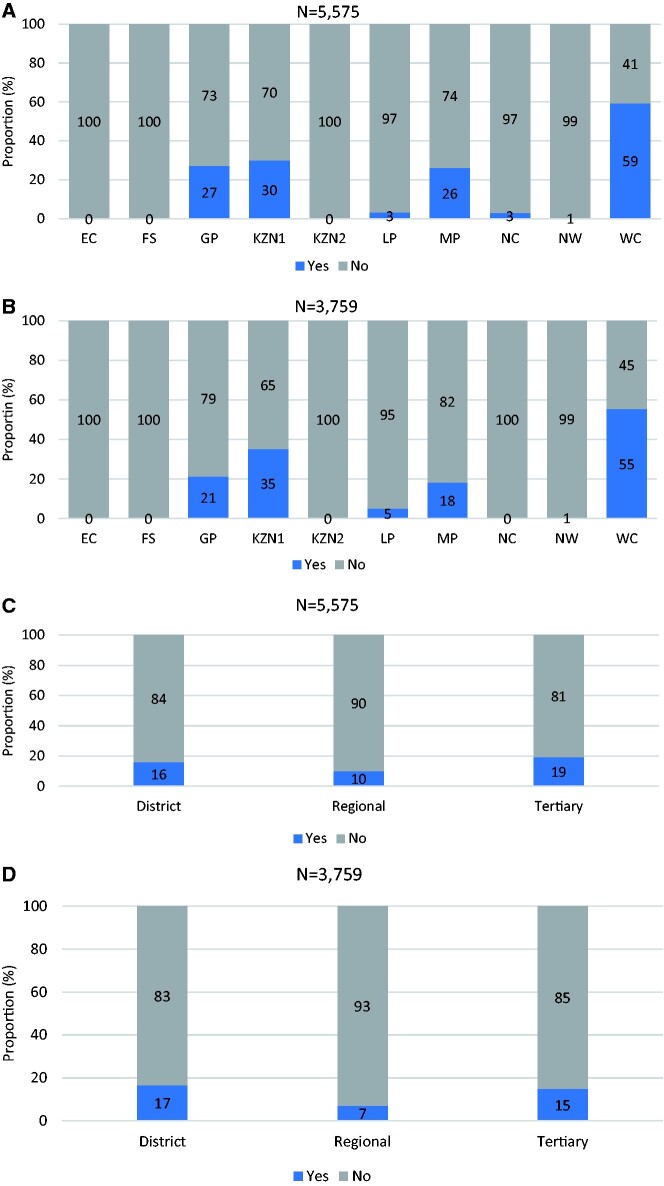
(a) Proportion of diagnoses coded using ICD-10 by NHI pilot district. (b) Proportion of diagnoses coded using ICD-10 by NHI pilot district for records with a discharge summary. (c) Proportion of diagnoses coded using ICD-10 by hospital type in the patient folder. (d) Proportion of diagnoses coded using ICD-10 by hospital type in discharge summaries.

The pilot district in the Western Cape province was the best-performing district with an estimated 59% (332/559) of patient folders including coded diagnoses (Figure 3a), but only 55% (250/452) of the available discharge summaries included coded diagnoses. Regarding hospital types, the best-performing hospitals were tertiary-level hospitals with 19% (160/825) of the patient folders including coded diagnoses, and a lower rate of diagnosis coding (15%; 74/491) in discharge summaries. Only 16% (533/3334) of patient folders and 17% (389/2338) of discharge summaries in district hospitals included coded diagnoses. The regional hospitals recorded the lowest rates of coded diagnoses: in 10% (142/1416) and 7% (66/930) of patient folders and discharge summaries respectively (Figure 3b).

### Accuracy of patient clinical records

In terms of the accuracy of the clinical records, Fleiss’ Kappa (*κ*) was used on four pre-defined data elements relevant to NHI reimbursement to compare three sources: ward registers, patients’ folders and discharge summaries (Tables 6 and 7). Across the three sources, the accuracy of recording of discharge date was only moderate (κ: 0.70; 95% CI 0.68–0.72). The accuracy of the rate at which physicians signed these documents (κ: 0.77; 95% CI 0.75–0.79) and the recording of the diagnosis during admission (κ: 0.77; 95% CI 0.74–0.80) was substantial. Accuracy of patients’ identification (κ: 0.90; 95% CI 0.89–0.92) in these sources was very good (Table 7).

**Table 6:** Measure of agreement between registers and patient folders

Variable	Register vs patient folders (*n* = 5795)					
	Agreement (%)	95% CI	Cohen’s Kappa (*κ*)	95% CI	*P*-value	Strength of agreement
Patient identifier	97.0	96.0–98.0	0.93	0.92–0.94	<0.001	Very good
Attending physician’s signature	85.0	84.0–86.0	0.70	0.68–0.72	<0.001	Substantial
Admission diagnosis	95.2	94.6–95.7	0.90	0.89–0.91	<0.001	Very good
Discharge date	81.3	80.3–82.3	0.62	0.59–0.64	<0.001	Moderate

**Table 7:** Measure of agreement between registers, patient folders and discharge summaries

Variable	Register vs patient folders vs discharge summary (*n* = 3767)					
	Agreement (%)	95% CI	Fleiss’ Kappa (*κ*)	95% CI	*P*-value	Strength of agreement
Patient identifier	96.0	95.0–97.0	0.90	0.89–0.92	<0.001	Very good
Attending physician’s signature	87.6	88.0–89.6	0.77	0.75–0.79	<0.001	Substantial
Admission diagnosis	89.3	88.5–90.1	0.77	0.74–0.80	<0.001	Substantial
Discharge date	85.7	84.8–86.6	0.70	0.68–0.72	<0.001	Moderate

We also used Cohen’s Kappa (*κ*) on 10 pre-defined data elements relevant to NHI reimbursement to compare two sources: patients’ folders and discharge summaries (Table 8). Discharge date was the least performing and showed only moderate agreement (*κ*: 0.60; 95% CI 0.57–0.63) between the data sources. Substantial agreement was achieved with patient’s age (*κ*: 0.72; 95% CI 0.70–0.74), condition on discharge (*κ*: 0.76; 95% CI 0.73–0.79); results of investigation (*κ*: 0.71; 95% CI 0.69–0.74); attending physician’s signature (*κ*: 0.71; 95% CI 0.67–0.75); and, follow-up plan (*κ*: 0.74; 95% CI 0.72–0.76). Very good levels of agreement were achieved with the recording of patients’ identifier (*κ*: 0.92; 95% CI 0.91–0.93); admission diagnosis (κ: 0.89; 95% CI 0.86–0.91); discharge diagnosis (κ: 0.92; 95% CI 0.90–0.94); and procedure(s) (*κ*: 0.86; 95% CI 0.84–0.89).

**Table 8:** Measure of agreement between patient folders and discharge summaries

Variable	Patient folders vs Discharge summary (*n* = 3767)					
	Agreement (%)	95% CI	Cohen’s Kappa (*κ*)	95% CI	*P*-value	Strength of agreement
Patient age	73.0	71.3–74.7	0.72	0.70–0.74	<0.001	Substantial
Patient identifier	96.1	95.5–96.7	0.92	0.91–0.93	<0.001	Very good
Attending physician’s signature	85.1	82.9–87.2	0.71	0.67–0.75	<0.001	Substantial
Admission diagnosis	87.3	86.2–88.4	0.89	0.86–0.91	<0.001	Very good
Discharge date	81.4	78.8–83.9	0.60	0.57–0.63	<0.001	Moderate
Discharge diagnosis	96.2	95.2–97.2	0.92	0.90–0.94	<0.001	Very good
Condition on discharge	88.5	86.5–90.6	0.76	0.73–0.79	<0.001	Substantial
Procedure(s)	93.3	91.4–95.2	0.86	0.84–0.89	<0.001	Very good
Follow-up plan	88.2	87.1–89.5	0.74	0.72–0.76	<0.001	Substantial
Results of investigation	86.0	84.0–88.1	0.71	0.69–0.74	<0.001	Substantial

## Discussion

Our study revealed that in the NHI pilot districts, document and documentation standards, including the availability of discharge summaries were suboptimal (since 35% of patient records did not include discharge summaries and available discharge summaries were not always appropriately completed). The absence of discharge summaries and the omission of critical discharge summary information, such as discharge medications, principal diagnosis and results of investigations, have been identified in other studies (Kripalani *et al.*, 2007; Adeleke *et al.*, 2012; Lydon *et al.*, 2018). While there have been attempts to improve the practice of providing and improving the quality of discharge summaries by using more structured formats or computer-generated summaries with positive results in terms of completeness (Dinescu *et al.*, 2011), there is still room for improvement as electronic discharge summaries have also been shown to be incomplete in other settings (Callen *et al.*, 2008; Cresswell *et al.*, 2015). Factors such as insufficient integration into routine work processes, insufficient training on completing discharge summaries and lack of education on the importance of discharge summaries (Callen *et al.*, 2008) may be responsible for the absence and suboptimal accuracy of the discharge summaries found in this study. Dinescu *et al.* (2011) suggested that an ‘audit and feedback’ educational intervention on discharge summaries could improve their quality.

The absence of discharge summaries in the patients’ clinical records and the large proportion of discharge summaries without coded diagnoses found in this study presents a potential challenge regarding the reimbursement of patients intending to claim from the NHI. Since coding facilitates billing (Manchikanti *et al.*, 2011; Dyers *et al.*, 2016), the absence of coded patient diagnoses and procedures will result in patients leaving treatment facilities before finalizing the billing process, thus delaying and complicating the reimbursement process. For the NHI, Regional and Tertiary hospitals will be paid based on diagnosis-related groups (DRG) framework that represents fixed amounts for each hospital stay (South African National Department of Health, 2015; Dyers *et al.*, 2016). When a hospital treats a patient and spends less than the DRG payment, it makes a profit and it loses money when the hospital spends more than the DRG payment. Therefore, the absence of key information from discharge summaries and incomplete routine coding could have considerable financial and resource management consequences for hospitals (Stavem *et al.*, 2002).

The ICD-10 classification has been described as extremely complicated and expensive, incurring costs related to human resources, hardware and software, which have the potential to delay reimbursement (Manchikanti *et al.*, 2011). Dyers *et al.* (2016) reported in a study in South Africa of using an ICD-10 coding tool in an electronic patient discharge record that only ∼43% of study records included complete ICD codes, and recommended improved coding tools, training and oversight as interventions to be considered to improve coding quality. We conclude, that there are challenges affecting the quality of discharge summaries regarding the coding of diagnoses in this study, indicating that the ICD-10 classification process is not yet operating satisfactorily and will need to be strengthened (Matsoso and Fryatt, 2013; Dyers *et al.*, 2016).

The substantial mismatch of patient age between the patient’s health records, ward registers and discharge summary is worrisome. Firstly, patient age is used for the classification of the burden of disease, which, in turn, informs resource distribution and interventions. Secondly, insurance claims vary with the age of the patient; therefore, incorrect age identification can affect the accuracy of the payment of claims.

Our study revealed that the absence of the attending physician’s signature was commonplace: although 95% of discharge summaries included the attending physician’s signature, discharge summaries were available for only 66% of patients. Another study indicated that the ‘attending physician’s signature’ component is omitted in some of the discharge summaries they reviewed, with hip-fracture discharge summaries exhibiting the lowest and cancer discharge summaries having the highest inclusion rates (Kind and Smith, 2008; Adeleke *et al.*, 2012). The absence of the attending physician’s signature on discharge summaries is of significance as the discharge summaries cannot be considered as valid without a signature (South African National Department of Health, 2004; Adeleke *et al.*, 2012; Wimsett *et al.*, 2014), and thus cannot be processed for insurance reimbursements. Insurance companies request the originals of all the medical reports, discharge summaries, consultation sheets, bills and receipts. The absence of the attending physician’s signature on discharge summaries will increase the chances of the claim being rejected, thus prolonging the claims period.

Our study of the NHI pilot sites provides an important situational analysis regarding the potential challenges that limited availability of discharge summaries can pose to health insurance reimbursements for the NHI. Most studies on discharge summaries have been single-site and focused on specific hospital types (Adeleke *et al.*, 2012; Horwitz *et al.*, 2013; Wimsett et al., 2014; Al-Damluji *et al.*, 2015; Dyers *et al.*, 2016). In our study, we compared the availability and quality of discharge summaries across nine provinces and three different hospital types: district, regional and tertiary. Our findings indicate that, irrespective of the hospital type, incomplete discharge summaries are ubiquitous. These findings are not surprising following the observation by other authors that even the highest-performing hospitals struggle to maintain quality discharge summaries in terms of timeliness, transmission and content (Van Walraven and Weinberg, 1995; Kind and Smith, 2008; Were *et al.*, 2009; Horwitz *et al.*, 2013; Wimsett *et al.*, 2014; Al-Damluji *et al.*, 2015, Dyers *et al.*, 2016). Therefore, interventions to improve the practice of completing discharge summaries and the quality of information recorded in the discharge summaries should be considered for all hospital types at all levels of the health system in South Africa.

### Study limitations

We intended to review the records of inpatients admitted in the months of March and July of 2015. While the data collection was started in 2016, we were unable to complete data collection in 2016 because of funding challenges. Thus, the data collection took place from 2016 to 2019. Consequently, some of the records for the study period could not be found as they had been transferred to other storage facilities or misplaced owing to the long period lapsed.

Based on our sampling approach, we were meant to systematically obtain at least 20 inpatient folders from each of the treatment departments. In some instances, where some folders were missing, we compensated by adjusting the sampling. For example, if folder 18 was missing, we selected folder 19. Also, in instances where the number of admissions for that month was <20 for the department, all the available folders were included.

## Conclusion

The discharge summary is a major source of routine morbidity and treatment data required to support health systems and the reimbursement of health care. Clinical data to support the planned NHI should be available for all inpatients in public hospitals at all levels. Without even considering the availability and utilization of electronic patient-information systems, the absence of completed discharge summaries constitutes a major barrier to efficient patient management and for determining the value of a claim for reimbursements. Even a small proportion of omitted information in the patient-discharge summary is of concern as it may affect patient safety as well as NHI reimbursements.

The absence of coded inpatient diagnoses and identified data inaccuracies indicate that existing RHISs in public-sector hospitals in the NHI pilot districts are not yet able to sufficiently support reimbursements and resource management. Institutional capacity is needed to undertake diagnostic coding, improve documentation of patient’s health records and ensure that a standard discharge summary is completed for every discharged inpatient, especially in the context of the planned implementation of the NHI in South Africa.

## Supplementary data

Supplementary data are available at *Health Policy and Planning* online.

## Supplementary Material

czab008_Supp
