# Supplementary material for: Is the routine health information system ready to support the planned national health insurance scheme in South Africa?
Source: Health Policy Plan. 2021 Apr 2;36(5):639–50. doi: 10.1093/heapol/czab008 (PMC8173599; doi:10.1093/heapol/czab008)
Supplement: czab008_Supp [file czab008_supp.zip › Figure 1.docx]

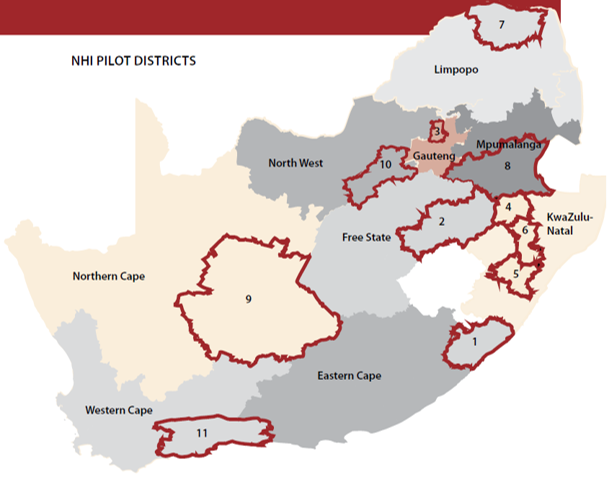


**Figure 1**: NHI pilot districts

1. OR Tambo; 2. Thabo Mofutsanyane; 3. City of Tshwane; 4. Amajuba; 5. uMgungundlovu; 6. uMzinyathi; 7. Vhembe; 8. Gert Sibanda; 9. Pixley ka Seme; 10. Dr Kenneth Kaunda; 11. Eden.
