# Supplementary material for: Is the routine health information system ready to support the planned national health insurance scheme in South Africa?
Source: Health Policy Plan. 2021 Apr 2;36(5):639–50. doi: 10.1093/heapol/czab008 (PMC8173599; doi:10.1093/heapol/czab008)
Supplement: czab008_Supp [file czab008_supp.zip › Figures 2A-C.docx]

**Fig 2A:** Proportion of available discharge summaries by NHI pilot district

**Fig 2B:**  Proportion of available discharge summaries by hospital type

**Fig 2C:** Proportion of available discharge summaries by hospital department
