# Supplementary material for: Is the routine health information system ready to support the planned national health insurance scheme in South Africa?
Source: Health Policy Plan. 2021 Apr 2;36(5):639–50. doi: 10.1093/heapol/czab008 (PMC8173599; doi:10.1093/heapol/czab008)
Supplement: czab008_Supp [file czab008_supp.zip › Figures 3A-D.docx]

**Fig 3A**: Proportion of diagnoses coded using ICD-10 by NHI pilot district

**Fig 3B**: Proportion of diagnoses coded using ICD-10 by NHI pilot district for records with a discharge summary

**Fig 3C**: Proportion of diagnoses coded using ICD-10 by hospital type in the patient folder

**Fig 3D**: Proportion of diagnoses coded using ICD-10 by hospital type in discharge summaries
