# Supplementary material for: Is the routine health information system ready to support the planned national health insurance scheme in South Africa?
Source: Health Policy Plan. 2021 Apr 2;36(5):639–50. doi: 10.1093/heapol/czab008 (PMC8173599; doi:10.1093/heapol/czab008)
Supplement: czab008_Supp [file czab008_supp.zip › Table 2.docx]

**Table 2**: Estimated numbers of records for review by types of public hospitals within NHI pilot districts

|  | **GP** | **WC** | **NC** | **NW** | **MP** | **FS** | **KZN1** | **KZN2** | **LP** | **EC** | **Total** |
| --- | --- | --- | --- | --- | --- | --- | --- | --- | --- | --- | --- |
| [**FACILITY TYPE**](file:///C:\Users\enicol\AppData\AppData\Local\Microsoft\Windows\Temporary%20Internet%20Files\AppData\Local\Microsoft\Windows\Temporary%20Internet%20Files\Content.MSO\536C32B4.xls#RANGE!A18) | **Tshwane** | **Eden** | **Pixley**  **ka Seme** | **Dr K Kaunda** | **Gert Sibande** | **Thabo Mofutsan-yane** | **uMgun-gundlovu** | **uMzin-yathi** | **Vhembe** | **O. R. Tambo** |  |
| District (Level 1) hospital | 3  (149)* | 3  (388) | 3  (578) | 2  (85) | 3  (506) | 3  (390) | 2  (222) | 3  (578) | 3  (430) | 3  (260) | **28**  **(3586)** |
| Regional (Level 2) hospital | 1  (64) | 1  (190) | 0  (0) | 2  (336) | 1  (72) | 2  (188) | 1  (255) | 0  (0) | 1  (148) | 1  (95) | **10**  **(1348)** |
| Tertiary/ central (Level 3) hospital | 3  (365) | 0  (0) | 0  (0) | 1  (156) | 0  (0) | 0  (0) | 1  (101) | 0  (0) | 0  (0) | 2  (224) | **7**  **(846)** |
| **Total** | **7**  **(578)** | **4**  **(578)** | **3**  **(578)** | **5**  **(578)** | **4**  **(578)** | **5**  **(578)** | **4**  **(578)** | **3**  **(578)** | **4**  **(578)** | **6**  **(578)** | **45**  **(5780)** |

*Estimated number of folders in parenthesis (Based on proportional sampling)

**GP:** Gauteng Province; **WC:** Western Cape; **NC:** Northern Cape; **MP:** Mpumalanga Province; **FS:** Free State Province; **KZN:** Kwa-Zulu Natal Province; **LP:** Limpopo Province; **EC:** Eastern Cape Province.
