# Supplementary material for: Is the routine health information system ready to support the planned national health insurance scheme in South Africa?
Source: Health Policy Plan. 2021 Apr 2;36(5):639–50. doi: 10.1093/heapol/czab008 (PMC8173599; doi:10.1093/heapol/czab008)
Supplement: czab008_Supp [file czab008_supp.zip › Table 3.docx]

**Table *3***: Overall response by facility type and treatment departments

| **Facility Type** | **Medicine** | **Surgery** | **Paediatrics** | **Obstetrics** | **Psychiatry** | **Total** |
| --- | --- | --- | --- | --- | --- | --- |
| District (Level 1) hospital  n (%) | 1,033  (29) | 696  (20) | 791  (22) | 873  (25) | 141  (4) | **3,532**  **(100)** |
| Regional (Level 2) hospital  n (%) | 280  (20) | 262  (18) | 351  (24) | 395  (28) | 149  (10) | **1,437**  **(100)** |
| Tertiary/Central (Level 3) hospital  n (%) | 220  (27) | 223  (27) | 137  (17) | 135  (16) | 111  (13) | **826**  **(100)** |
| Total  n (%) | **1,533**  **(27)** | **1,181**  **(20)** | **1,279**  **(22)** | **1,401**  **(24)** | **401**  **(7)** | **5,795**  **(100)** |
