# Supplementary material for: Is the routine health information system ready to support the planned national health insurance scheme in South Africa?
Source: Health Policy Plan. 2021 Apr 2;36(5):639–50. doi: 10.1093/heapol/czab008 (PMC8173599; doi:10.1093/heapol/czab008)
Supplement: czab008_Supp [file czab008_supp.zip › Table 4.docx]

**Table *4***: Response rate (Rr) showing overall proportion against the target by facility type, pilot district and province

|  | **GP** | **WC** | **NC** | **NW** | **MP** | **FS** | **KZN1** | **KZN2** | **LP** | **EC** | **Total** |
| --- | --- | --- | --- | --- | --- | --- | --- | --- | --- | --- | --- |
| [**FACILITY TYPE**](file:///C:\Users\enicol\AppData\AppData\Local\Microsoft\Windows\Temporary%20Internet%20Files\AppData\Local\Microsoft\Windows\Temporary%20Internet%20Files\Content.MSO\536C32B4.xls#RANGE!A18) | **Tshwane** | **Eden** | **Pixley**  **ka Seme** | **Dr K Kaunda*** | **Gert Sibande** | **Thabo Mofutsan-yane** | **uMgun-gundlovu** | **uMzin-yathi** | **Vhembe** | **O. R. Tambo** |  |
| District (Level 1) hospital  (n1/N, n2) | 3/3  (149) | 3/3  (388) | 3/3  (578) | 1/2  (85) | 3/3  (506) | 3/3  (390) | 2/2  (222) | 3/3  (578) | 3/3  (430) | 3/3  (260) | 27/28  (3,586) |
| **District Rr (%, n3)** | **98**  (146) | **105**  (409) | **100**  (578) | **92**  (78) | **95**  (483) | **99**  (389) | **93**  (207) | **100**  (580) | **97**  (418) | **98**  (256) | **99**  (3,544) |
| Regional (Level 2) hospital  (n1/N, n2) | 1/1  (64) | 1/1  (190) | 0  (0) | 2/2  (336) | 1/1  (72) | 2/2  (188) | 1/1  (255) | 0  (0) | 1/1  (148) | 1/1  (95) | 10/10  (1,348) |
| **Regional Rr (%, n3)** | **106**  (68) | **103**  (196) | **-** | **100**  (336) | **60**  (43) | **107**  (201) | **100**  (256) | **-** | **115**  (170) | **164**  (156) | **106**  (1,426) |
| Tertiary/ central (Level 3) hospital  (n1/N, n2) | 3/3  (365) | 0  (0) | 0  (0) | 1/1  (156) | 0  (0) | 0  (0) | 1/1  (101) | 0  (0) | 0  (0) | 2/2  (224) | 7/7  (846) |
| **Tertiary Rr (%, n3)** | **104**  (378) | **-** | **-** | **114**  (178) | **-** | **-** | **102**  (103) | **-** | **-** | **74**  (166) | **98**  (825) |
| Total | 7/7  (578) | 4/4  (578) | 3/3  (578) | 4/5  (578) | 4/4  (578) | 5/5  (578) | 4/4  (578) | 3/3  (578) | 4/4  (578) | 6/6  (578) | 44/45  (5,780) |
| **Total Rr**  **(%, n3)** | **102**  **(592)** | **105**  **(605)** | **100**  **(578)** | **102**  **(592)** | **91**  **(526)** | **102**  **(590)** | **98**  **(566)** | **100**  **(580)** | **102**  **(588)** | **100**  **(578)** | **100**  **(5,795)** |

*n1 – number of targeted facilities; N – total number of available facilities; n2 – estimated number of folders & n3 – number of folders obtained*

* Dr K Kaunda district only has two district level hospitals, of which one was downgraded to a Community Health Centre and therefore no longer met the sampling criteria.
