# Supplementary material for: Is the routine health information system ready to support the planned national health insurance scheme in South Africa?
Source: Health Policy Plan. 2021 Apr 2;36(5):639–50. doi: 10.1093/heapol/czab008 (PMC8173599; doi:10.1093/heapol/czab008)
Supplement: czab008_Supp [file czab008_supp.zip › Table 5.docx]

**Table *5***: Document and documentation standards

| **Detail** | **n/N**** | **Percentage: 95% CI** |
| --- | --- | --- |
| ***Clinical detail and quality assurance – document standards*** | | |
| Discharge notes are recorded in progress notes on discharge | 4,454/5,790 | 77 (70.8-83.1)***** |
| Follow-up details are written as part of the discharge notes | 3,749/5,787 | 65 (55.4-74.1)***** |
| Standard medical record includes a discharge summary | 3,767/5,743 | 66 (56.1-75.0)***** |
| Clerking and follow-up notes from admission to discharge | 5,145/5,789 | 89 (84.7-93.1) |
| Discharge summary completed by a clinician | 3,695/3,884 | 95 (91.1-99.2) |
| ***Data reliability, consistency and responsibility for care – documentation standards*** | | |
| All pages contain patient’s full names | 3,722/5,788 | 64 (55.6-73.0)***** |
| Patient identifier recorded in all pages | 3,607/5,790 | 62 (53.8-70.8)***** |
| All pages contain correct patient identification | 3,664/5,791 | 63 (54.5-72.0)***** |
| Progress notes from admission to discharge | 4,926/5,789 | 85 (80.4-89.8) |
| Progress notes documented daily | 4,939/5,789 | 85 (80.6-90.0) |
| Notes signed and dated daily | 5,024/5,789 | 87 (82.5-91.0) |

*****Elements that did not meet the exception rate of ±20% tolerance levels (i.e. the permissible range of variation) within expected values. ****** Variation in N (denominator) as a result of missing data
