# Supplementary material for: Is the routine health information system ready to support the planned national health insurance scheme in South Africa?
Source: Health Policy Plan. 2021 Apr 2;36(5):639–50. doi: 10.1093/heapol/czab008 (PMC8173599; doi:10.1093/heapol/czab008)
Supplement: czab008_Supp [file czab008_supp.zip › Tables 6a-c.docx]

**Table 6a:** Measure of agreement between registers and patient folders

| **Variable** | **Register vs Patient folders (n=5,795)** | | | | | |
| --- | --- | --- | --- | --- | --- | --- |
|  | **Agreement (%)** | **95% CI** | **Cohen’s Kappa (*κ*)** | **95% CI** | **p-value** | **Strength of agreement** |
| Patient identifier | 97.0 | 96.0-98.0 | 0.93 | 0.92-0.94 | <0.001 | Very good |
| Attending physician’s signature | 85.0 | 84.0-86.0 | 0.70 | 0.68-0.72 | <0.001 | Substantial |
| Admission diagnosis | 95.2 | 94.6-95.7 | 0.90 | 0.89-0.91 | <0.001 | Very good |
| Discharge date | 81.3 | 80.3-82.3 | 0.62 | 0.59-0.64 | <0.001 | Moderate |

**Table 6b:** Measure of agreement between registers, patient folders and discharge summaries

| **Variable** | **Register vs Patient folders vs Discharge summary (n=3,767)** | | | | | |
| --- | --- | --- | --- | --- | --- | --- |
|  | **Agreement (%)** | **95% CI** | **Fleiss’ Kappa (*κ*)** | **95% CI** | **p-value** | **Strength of agreement** |
| Patient identifier | 96.0 | 95.0-97.0 | 0.90 | 0.89-0.92 | <0.001 | Very good |
| Attending physician’s signature | 87.6 | 88.0-89.6 | 0.77 | 0.75-0.79 | <0.001 | Substantial |
| Admission diagnosis | 89.3 | 88.5-90.1 | 0.77 | 0.74-0.80 | <0.001 | Substantial |
| Discharge date | 85.7 | 84.8-86.6 | 0.70 | 0.68-0.72 | <0.001 | Moderate |

**Table 6c:** Measure of agreement between patient folders and discharge summaries

| **Variable** | **Patient folders vs Discharge summary (n=3,767)** | | | | | |
| --- | --- | --- | --- | --- | --- | --- |
|  | **Agreement (%)** | **95% CI** | **Cohen’s Kappa (*κ*)** | **95% CI** | **p-value** | **Strength of agreement** |
| Patient age | 73.0 | 71.3-74.7 | 0.72 | 0.70-0.74 | <0.001 | Substantial |
| Patient identifier | 96.1 | 95.5-96.7 | 0.92 | 0.91-0.93 | <0.001 | Very good |
| Attending physician’s signature | 85.1 | 82.9-87.2 | 0.71 | 0.67-0.75 | <0.001 | Substantial |
| Admission diagnosis | 87.3 | 86.2-88.4 | 0.89 | 0.86-0.91 | <0.001 | Very good |
| Discharge date | 81.4 | 78.8-83.9 | 0.60 | 0.57-0.63 | <0.001 | Moderate |
| Discharge diagnosis | 96.2 | 95.2-97.2 | 0.92 | 0.90-0.94 | <0.001 | Very good |
| Condition on discharge | 88.5 | 86.5-90.6 | 0.76 | 0.73-0.79 | <0.001 | Substantial |
| Procedure(s) | 93.3 | 91.4-95.2 | 0.86 | 0.84-0.89 | <0.001 | Very good |
| Follow-up plan | 88.2 | 87.1-89.5 | 0.74 | 0.72-0.76 | <0.001 | Substantial |
| Results of Investigation | 86.0 | 84.0-88.1 | 0.71 | 0.69-0.74 | <0.001 | Substantial |
