# Supplementary material for: Is the routine health information system ready to support the planned national health insurance scheme in South Africa?
Source: Health Policy Plan. 2021 Apr 2;36(5):639–50. doi: 10.1093/heapol/czab008 (PMC8173599; doi:10.1093/heapol/czab008)
Supplement: czab008_Supp [file czab008_supp.zip › Table_1.docx]

**Table 1**: Characteristics of the different hospital levels (NDoH, 2004)

| **Care level** | **Characteristics of hospital type** | **Total** | **Number sampled** |
| --- | --- | --- | --- |
| Tertiary hospitals (Level 3) | - Has between 400 – 800 beds - Provides specialist services - Provides intensive care services under the supervision of a specialist - Receives referrals from regional and district hospitals without provincial boundaries | 7 | 7 |
| Regional hospitals (Level 2) | - Has between 400 – 800 beds - Provides specialist services on a 24-hour basis - Receives outreach support from tertiary hospitals | 10 | 10 |
| District hospitals (Level 1) | - Serves a defined population within a district and supports primary health care. - Can have from 50 – 600 beds depending on the size - Provides district package of care on a 24-hour basis - General practitioners and clinical nurse practitioners providing health services. - Provides in-patient, emergency and ambulatory health services. | 51 | 28* |
| **Total hospitals** | | **68** | **45** |

* There were only two district hospitals in two of the districts (Dr K Kaunda and uMgungundlovu)
