# Supplementary material for: Is the routine health information system ready to support the planned national health insurance scheme in South Africa?
Source: Health Policy Plan. 2021 Apr 2;36(5):639–50. doi: 10.1093/heapol/czab008 (PMC8173599; doi:10.1093/heapol/czab008)
Supplement: czab008_Supp [file czab008_supp.zip › Appendix A_MbHIS EVAL data collection framework.doc]

***Appendix A: MbHIS-EVAL – DATA QUALITY STUDY – data collection summary checklist***

***Hospital: ________ Northdale Hospital ___________Date of data collection ___________________ Data collector _______________***

| **S/N** | **Patient study ID** | **Folder number (ID)**  **(Register)** | **Paper** | | | **Ward/ Department** | ***Patient folder (selected)*** | **Electronic** | | | **Comment** |
| --- | --- | --- | --- | --- | --- | --- | --- | --- | --- | --- | --- |
|  |  |  | **Ward register PHOTO** | **Patient folder PHOTO** | **Discharge summary PHOTO** |  |  | **Ward register** | **Patient folder/ record** | **Discharge summary** |  |
| 1. | NTDKZN001 |  |  |  |  |  |  |  |  |  |  |
| 2. | NTDKZN002 |  |  |  |  |  |  |  |  |  |  |
| 3. | NTDKZN003 |  |  |  |  |  |  |  |  |  |  |
| 4. | NTDKZN004 |  |  |  |  |  |  |  |  |  |  |
| 5. | NTDKZN005 |  |  |  |  |  |  |  |  |  |  |
| 6. | NTDKZN006 |  |  |  |  |  |  |  |  |  |  |
| 7. | NTDKZN007 |  |  |  |  |  |  |  |  |  |  |
| 8. | NTDKZN008 |  |  |  |  |  |  |  |  |  |  |
| 9. | NTDKZN009 |  |  |  |  |  |  |  |  |  |  |
| 10. | NTDKZN010 |  |  |  |  |  |  |  |  |  |  |
| 11. | NTDKZN011 |  |  |  |  |  |  |  |  |  |  |
| 12. | NTDKZN012 |  |  |  |  |  |  |  |  |  |  |
| 13. | NTDKZN013 |  |  |  |  |  |  |  |  |  |  |
| 14. | NTDKZN014 |  |  |  |  |  |  |  |  |  |  |
| 15. | NTDKZN015 |  |  |  |  |  |  |  |  |  |  |
| 16. | NTDKZN016 |  |  |  |  |  |  |  |  |  |  |
| 17. | NTDKZN017 |  |  |  |  |  |  |  |  |  |  |
| 18. | NTDKZN018 |  |  |  |  |  |  |  |  |  |  |
| 19. | NTDKZN019 |  |  |  |  |  |  |  |  |  |  |
| 20. | NTDKZN020 |  |  |  |  |  |  |  |  |  |  |

***Signature of data collector __________________	Date of submission __________________	Time spent at facility (hr) __________________***
